# AI as the Interpreter for Identifying Root Causes and Emotional Themes in Mental Health Narratives on Reddit Using AutoML and PaLM 2: Mixed Methods Study

**DOI:** 10.2196/71219

**Published:** 2026-07-13

**Authors:** Saima R, Khandakar Ahmed, Manjula O’Connor

**Affiliations:** 1 Institute for Sustainable Industries and Liveable Cities (ISILC), Victoria University, 70–104 Ballarat Road, Footscray, Melbourne, Victoria, 3011, Australia, 61 (03) 9919 6100; 2 Department of Psychiatry, University of Melbourne, Melbourne, Australia

**Keywords:** mental health, social media, artificial intelligence, machine learning, natural language processing, interpretability, contextual reasoning, text classification

## Abstract

**Background:**

Mental health issues continue to increase worldwide, often intensified by stigma and lack of awareness. Social media has become a major space where individuals articulate their emotional and psychological experiences. However, little is known about how AI models interpret these narratives, particularly when contextual reasoning and alignment with human judgment are required.

**Objective:**

This study examines 2 AI models, AutoML and PaLM 2, to classify mental health root causes in social media posts and evaluate their alignment with human judgment. The aim is to assess AI-human agreement and model interpretability when applied to complex, real-world mental health narratives.

**Methods:**

AutoML and PaLM 2 were trained to classify mental health root causes using an annotated dataset of Reddit (Reddit, Inc.) posts (n=800). Model generalizability and alignment with human judgment were evaluated on a hold-out set of unseen posts (n=50) using human consensus labels. Quantitative analyses included AI-human agreement assessment and statistical comparison of paired predictions. Qualitative analyses examined misclassification patterns, contextual reasoning, and interpretability using a structured coding approach.

**Results:**

On the unseen posts, PaLM 2 aligned with human consensus on 40/50 (80%) posts, while AutoML aligned on 34/50 (68%) posts. Agreement between PaLM 2 and human raters (Cohen κ≈0.72) fell within a similar range to that observed for AutoML (Cohen κ≈0.67). In the paired comparison of model errors, the McNemar test based on discordant pairs (PaLM 2 correct and AutoML incorrect: 12; AutoML correct and PaLM 2 incorrect: 6) did not indicate a difference in classification outcomes between models (*χ*²_₁_=1.39, *P*=.24). Qualitative analysis identified differences in the output patterns of the models when processing contextual and emotional information, with PaLM 2 often generating outputs consistent with broader narrative context and AutoML relying more heavily on lexical features.

**Conclusions:**

AutoML showed strong internal performance yet reduced generalizability to unseen mental health narratives. PaLM 2 demonstrated distinct alignment patterns relative to human consensus and outputs consistent with greater sensitivity to narrative context, although both models did not differ significantly in error distribution. Both models and human raters struggled with inherently ambiguous categories, underscoring the complexity of mental health discourse. These findings highlight the value of combining quantitative and interpretability-focused analysis to advance transparent AI for mental health text classification.

## Introduction

The global surge in mental health issues poses a serious challenge to public health systems. This is further complicated by widespread stigma and lack of awareness [1]. Such underrecognition and underdiagnosis patterns become a hindrance to effective treatment and timely support [2,3]. As a result, the alarming rise in mental health disorders significantly increases the risk of suicide [4,5]. In Australia, for instance, mental health issues affect half of the population, contributing to approximately 3000 suicides annually [6]. From 2011 to 2021, suicide rates among males and females increased from 16.2 to 18.6 and from 5.1 to 5.8 deaths per 100,000 individuals, respectively [7]. These numbers explain the urgent need for effective treatment strategies to alleviate both human and economic burdens on health care systems [8,9].

Mental health disorders significantly contribute to nonsuicidal self-injury and suicidal behaviors [10,11]. However, the transition from suicidal ideation to attempts is poorly understood, marked by a stark disparity between the high prevalence of mental disorders and the lower incidence of suicide attempts [12,13]. This gap underlines the difficulty in predicting and intervening in suicidal behaviors, which is a critical area of concern for mental health professionals [14].

In this digital age, social media platforms serve as crucial venues for individuals to express their mental health struggles, turning these platforms into valuable resources for early detection and intervention of mental health issues [15]. The extensive data generated on these platforms necessitate advanced, automated methods for effective analysis due to the impracticality of manual processing [16,17]. While machine learning shows promise, the field urgently requires innovative approaches to decipher the root causes behind mental health conditions expressed on social media [18,19]. Despite some progress [20], a significant gap remains in identifying risk factors that could enhance predictive capabilities [21-23].

The identification of mental health root causes is an area that necessitates thorough evaluation and transparency regarding predictive outcomes. Existing studies are often limited to a handful of binary mental health detection tasks and lack interpretability in their findings [24,25]. Many of these efforts rely on simplistic prompt-based approaches [26,27], thereby neglecting the rich emotional context embedded in user-generated narratives. Yet, emotional indicators have proven to be instrumental in prior mental health analysis [28].

For instance, Garg et al [25] introduced valuable datasets and annotation schemas for causal analysis, primarily emphasizing corpus creation and baseline model performance. Similarly, our previous work [29] developed a root cause–annotated dataset of Reddit (Reddit, Inc.) posts. However, neither explored how AI models interpret mental health narratives or align with human reasoning. Understanding these interpretive processes remains an important but underexamined area.

This study addresses this gap by evaluating AutoML and PaLM 2 on their ability to classify root causes of mental health narratives from Reddit [30]. We used the annotated dataset from our previous study [29] to train and evaluate these models in terms of both predictive performance and interpretability [31,32]. We further analyze the extent to which these models incorporate contextual and emotional cues and how closely their outputs align with human consensus beyond surface-level accuracy [33].

To investigate this, we use a 2-phase research approach. First, both models are trained on the annotated dataset. Second, the models classify unseen posts into 4 specific root causes. We then compare their predictions with human raters’ judgments to evaluate model-human alignment in interpreting complex psychological content [30,34].

We hypothesize that AI may assist in the preliminary analysis of mental health narratives by identifying patterns that could support human assessment [35]. Differences in interpretability between models highlight where AI aligns with or diverges from human interpretive tendencies [36]. These divergences offer insight into strengths, limitations, and areas requiring refinement for improved processing of contextual information.

The aim of this study is to assess the extent to which AutoML and PaLM 2 can generalize to unseen mental health narratives and evaluate how their predictions and output patterns align with human interpretations. By analyzing model-human agreement, label-specific misclassification patterns, and qualitative reasoning, this work contributes to ongoing efforts in mental health informatics by examining not only what these models predict but also how they interpret narrative content [37].

## Methods

### Datasets

Reddit is well known for its diverse and anonymous user base in the research industry [38,39]. It provides a distinctive opportunity to investigate the complex discourse related to mental health issues. We used the Reddit Mental Health Dataset (RMHD) from our previous study [29], which includes over 1 million posts collected from 5 key subreddits related to mental health (r/anxiety, r/depression, r/mentalhealth, r/SuicideWatch, and r/lonely) between January 2019 and August 2022. Posts were retrieved using the Pushshift application programming interface in accordance with Reddit’s Terms of Service. Due to Reddit’s anonymous nature, no demographic or geolocation data were collected.

We further divide RMHD into 2 distinct subsets: the Mental Health-Reddit Annotated Corpus (MH-RAC) and RMHD-Seed. These subsets were created to facilitate focused examination of linguistic and emotional nuances in mental health narratives.

### Ethical Considerations

The original data collection was approved by Victoria University Human Research Ethics Committee (ID: HRE23-005), consistent with the National Health and Medical Research Council (NHMRC) National Statement on Ethical Conduct in Human Research (2007, updated 2018).

All data consisted of publicly available Reddit posts and were handled in a fully deidentified form. User names, URLs, and any personal identifiers were excluded prior to analysis. The dataset includes first-person mental health narratives, some of which reference psychological distress; these posts were retained to preserve contextual and semantic integrity. No attempt was made to identify, track, or intervene with individual users.

### MH-RAC

MH-RAC (n=800) is an annotated subset created and validated in our previous work [29] and reused in this study as the primary labeled dataset for model training, evaluation, and interpretability analysis. In this study, no new annotation was performed on MH-RAC; however, the same finalized annotation guidelines from our previous study [29] were reused to guide human rater label assignment on RMHD-Seed, ensuring consistency between ground truth labels and the evaluation procedure.

MH-RAC was constructed from RMHD using a multistage selection process. Posts were first sourced from mental health–focused subreddits to ensure topical relevance, then identified via keyword-assisted screening aligned with predefined root-cause concepts to identify candidate posts. Each selected post was subsequently reviewed in full to confirm that the narrative contained sufficient contextual evidence of a single dominant root cause. Posts reflecting multiple competing root causes were excluded to preserve single-label interpretability. This structured, multistage selection process ensured deliberate coverage across the 4 predefined mental health root-cause categories.

Posts were labeled using a deductive approach, whereby each post was assigned to 1 of 4 predefined mental health root cause categories reflecting prominent psychological determinants described by Health Direct Australia [40]:

1. Drugs and alcohol: posts referring to substance use as a contributing factor to mental distress or disorders.
2. Early life: posts describing early childhood experiences (eg, neglect, abuse, and unstable environments) as precursors to mental health issues.
3. Personality: posts linking personal traits such as introversion, overthinking, or low self-esteem to mental health challenges.
4. Trauma and stress: posts recounting recent or past traumatic events, chronic stress, or high-pressure life events.

Each category contains 200 posts following annotation, yielding a balanced 4-class dataset. This balanced design was used to reduce class-imbalance effects during supervised learning and to enable category-level comparison of model errors and human-AI alignment.

The annotation protocol in the study by Rani et al [29] included written guidelines, iterative annotator training and calibration with domain expert input, independent double-coding, and consensus-based adjudication of disagreements. Interannotator agreement for the 800 posts was quantified in our previous study [29] using Cohen κ=0.869, indicating very high agreement.

All annotated posts represent first-person narratives in which users describe their own thoughts, experiences, or emotional challenges, supporting thematic consistency for both model training and interpretability analyses.

### RMHD-Seed

The RMHD-Seed (n=50) comprises posts held out from the MH-RAC and excluded from all training and validation stages to preserve an unbiased evaluation set.

Although small, the set was intentionally designed to be representative and analytically manageable, enabling precise model inference and a direct comparison with human ratings on identical data.

### Mental Health Evaluation Corpus

We used RMHD-Seed to construct the Mental Health Evaluation Corpus (MHEC). Both trained models generated predicted labels for all RMHD-Seed posts, and the same posts were independently labeled by 3 human raters. MHEC thus contains, for each post, the human consensus label and the corresponding predictions from AutoML and PaLM 2. This corpus served as the basis for evaluating model-human agreement, misclassification patterns, and interpretability.

### Model Training

MH-RAC underwent standard text preprocessing, including text cleaning, stop word removal, and tokenization. We selected Google AI’s PaLM 2 model due to its robust natural language understanding capabilities and suitability for sensitive mental health data. We also include Google’s AutoML model for its automated optimization. We use Google Cloud’s Vertex AI platform as an all-inclusive environment for model training and deployment and provide us with more flexibility with our data.

### AutoML

AutoML approaches automate model selection and hyperparameter optimization [41,42]. We used Google Cloud AutoML, which is a managed training service for automated model development via Vertex AI AutoML Text Classification, to train a single-label, multiclass text classifier on the MH-RAC dataset.

For training, MH-RAC was converted into JSON Lines (JSONL) format to adhere to the AutoML’s required schema (Textbox 1). This ensured the correct parsing of both text and labels for model training.

Textbox 1. **JSONL schema for AutoML input format.**

{

“classificationAnnotation”: {

“displayName”: “Root Cause Label”

},

“textContent”: “Reddit Post text,”

“dataItemResourceLabels”:

{

“aiplatform.googleapis.com/ml_use”: “training/test/validation”

}

}

In this schema, the “displayName” attribute holds the annotated label, and the “textContent” field stores the Reddit post text. The “dataItemResourceLabels” tag specifies the dataset role, enabling AutoML to automatically stratify the data into an 80/10/10 train-validation-test split.

Training was conducted in the “us-central1” region and completed in approximately 15 hours. The task objective was single-label text classification, and model training and evaluation were performed entirely within AutoML’s managed pipeline.

AutoML automatically handled feature extraction, model selection, and internal hyperparameter optimization using its default configuration. User-configurable settings were limited to dataset specification. Low-level hyperparameters such as learning rate, batch size, number of epochs, and model architecture are not exposed by the platform and therefore cannot be directly reported. All model hyperparameters were determined automatically by the AutoML system, and no manual hyperparameter tuning was performed by the authors. Model performance was evaluated using AutoML’s internal validation and test sets.

### PaLM 2

Google’s PaLM 2 is an advanced foundation model designed for high-performance NLP tasks, including classification and contextual reasoning. We used the PaLM 2 for Text (text-bison@001) model via Vertex AI. As a proprietary, closed-source system, full architectural reproducibility is limited to users within the Google Cloud environment. Fine-tuning was conducted using the managed Vertex AI “Model Tuning” service.

### Model Configuration and Fine-Tuning

PaLM 2 was instruction-tuned on the MH-RAC dataset to improve its contextual alignment with mental health language [43]. Fine-tuning was performed via the Vertex AI “Model Tuning” service using Parameter-Efficient Fine-Tuning.

MH-RAC was formatted into JSON Lines (JSONL) to match text-bison@001’s input-output schema (Textbox 2). Each entry contained an “input_text” field which was structured as a classification instruction (eg, “Classify the following text...”) followed by the Reddit post and an “output_text” field containing the correct label. During inference, the same prompt structure was used without the label to obtain model predictions.

Textbox 2. **Training data schema for PaLM2 text-bison001.**

{

“input_text”: “Classify the following text into one of the following classes: [drugsalcohol, earlylife, Personality, trauma] Text: {input_text},”

“output_text”: “Corresponding mental health Root Cause label”

}

The system adheres to the model’s 8192-token context window, allowing entire posts to be passed as full-context prompts.

The training set consisted of 560 posts, with 240 held for validation, while RMHD-Seed was held out entirely for final evaluation against human consensus. The tuning regime was configured for 100 steps with a default batch size of 8, resulting in approximately 1.43 epochs over the training data. The learning rate multiplier was set to 1.0. These settings were selected to prioritize stability and prevent overfitting given the specialized, relatively small-scale nature of the MH-RAC dataset. Model optimization followed the standard cross-entropy loss provided by the supervised tuning pipeline.

### Inference Reproducibility and Prompting Strategy

During the evaluation on the RMHD-Seed dataset, inference was performed using instruction-based prompts applied to the fine-tuned model. Prompts were example-free and consisted only of the classification instruction and the raw text, without any in-context examples.

To ensure total methodological transparency and inference reproducibility, we strictly enforced a deterministic environment using the following hyperparameters:

- Temperature: 0 (to eliminate randomness)
- Top-K: 1 and Top-P: 0 (forcing the model to select only the single highest probability token)
- Max output tokens: 2 (to ensure high-velocity classification and constrain outputs to a single class label)

Chain-of-thought or rationale outputs were not requested or collected; the model was constrained to generate only the final class label. All inferences were performed using a standardized prompt template consistent with the fine-tuning schema (Textbox 2).

### Evaluation Framework

The evaluation framework provides a basis for comparing AI model performance with human raters. This enables a systematic assessment of how each model classifies mental health narratives and the extent to which their outputs align with human judgment.

### Human Raters Team Composition

The team consisted of 3 raters to capture a comprehensive range of interpretative perspectives [44]. Rater 1, as domain expert, played an integral role in the evaluation process [45], providing deep expertise to interpret mental health narratives.

Rater 2 was a trained annotator from the research team and familiar with the root-cause annotation guidelines [46]. Rater 3 was a layperson, representing the viewpoint of an informed nonexpert and contributing a perspective closer to that of the public, which was crucial for enhancing the accessibility and relevance of interventions [30].

Together, the raters provided a balanced mix of domain knowledge, methodological consistency, and everyday interpretive judgment.

### Human Rater Label Assignment

The label assignment process was designed to uphold methodological rigor and maintain consistency with the root-cause annotation guidelines used for the MH-RAC dataset. RMHD-Seed posts were anonymized to maintain confidentiality. Each rater independently assigned 1 of the 4 root-cause labels to every post, guided by standardized written instructions aligned with the MH-RAC annotation protocol to minimize evaluation bias. No additional training sessions were required, as the protocol had been previously established [29]. To preserve the integrity of their individual interpretative processes, each rater performs the task in isolation to prevent interrater influence. They are also required to provide evaluative rationale with their classifications to understand their perspective.

### AI-Driven Label Assignment

After training, both models were deployed via Vertex AI for inference. AutoML and PaLM 2 each classified all RMHD-Seed posts, generating 1 of the 4 root-cause labels per post. These outputs were subsequently compared with human consensus to evaluate agreement, misclassification patterns, and interpretability.

### Consensus Benchmark

For each RMHD-Seed post, we derived a consensus label using majority agreement. A post was assigned a final label when at least 2 of 3 raters selected the same category. This majority rule produced a single consensus label per post, which served as the reference standard for evaluating AI model predictions.

### Analysis Approach

Our analysis integrated quantitative agreement metrics with qualitative interpretability assessment to examine model behavior relative to human judgment.

### Interrater Reliability Analysis

We assessed consistency among 3 human raters using Cohen κ and extended the same measure to compare each AI model (PaLM 2 and AutoML) with human raters by treating the models as additional quasi-raters. This approach provided a unified agreement matrix capturing reliability and alignment across all raters.

### Statistical Comparison

We used the McNemar test to assess whether AutoML and PaLM 2 differed in classification outcomes on the same set of unseen posts. For each post, predictions from both models were coded as correct or incorrect relative to the human consensus label. A 2×2 contingency table of paired outcomes was constructed, focusing on discordant prediction pairs (ie, cases where one model was correct and the other incorrect). A 2-sided McNemar test with continuity correction was applied to evaluate differences in paired error rates, using a significance threshold of α=.05.

### Label-Specific Misclassification Analysis

We examined misclassification frequencies for each label to understand where and how the models diverged from human consensus. This label-level error analysis quantified how often each model misassigned posts and highlighted categories that were particularly challenging (eg, early life vs trauma and stress). These patterns provided a fine-grained basis for comparing PaLM 2 and AutoML.

### Contextual and Explainability Analysis

We conducted a 2-pronged analytical approach to investigate how AI models interpret complex narratives and align with human judgment.

#### Contextual Analysis Based on Consensus Rationale

##### Overview

We compared model predictions with the consensus labels and corresponding human rationales to examine how well each model captured thematic and semantic meaning. This analysis focused on areas of alignment and divergence in handling contextual cues and overlapping life events.

##### Lexical Versus Thematic Error Analysis

We identified narrative factors that contributed to classification confusion and assessed whether these discrepancies stemmed from surface-level lexical reliance or limitations in capturing broader thematic context from the text. This analysis evaluated how closely each model’s classification outputs corresponded with human thematic judgments and highlighted cases in which lexical cues led to incorrect classifications.

##### Explainability Analysis Using Interpretability Coding Schema

To examine model reasoning behaviors, we developed a study-specific interpretability schema to describe observable patterns in model-generated outputs. The schema was designed to support qualitative examination of how models appeared to arrive at their predictions in selected illustrative cases. Rather than assessing internal model mechanisms or feature attribution processes, the analysis focused on reasoning cues reflected in the textual explanations produced by the models. This perspective is consistent with human-centered approaches to evaluating explanation artifacts in explainable AI research [47].

This schema captures five reasoning patterns:

1. Keyword reliance (lexical anchoring): decisions based primarily on isolated lexical cues.
2. Context integration (narrative alignment): use of multisentence context, temporal progression, or synthesis of multiple life events within the narrative.
3. Emotional cue recognition (affective correspondence): attention to emotional tone, crisis language, or explicit expressions of distress reflected in the text.
4. Ambiguity and overlap handling (multifactor sensitivity): navigation of narratives with overlapping root causes.
5. Causal reasoning (root-cause association): outputs associating the narrative with a dominant contributing factor related to mental distress.

For selected illustrative instances, including both correct classifications and misclassifications, we identified the dominant reasoning pattern reflected in each model’s output. The framework was applied as a descriptive analytic tool to support qualitative interpretation of model behavior rather than as a formal qualitative coding study intended to produce generalizable coding frequencies. Coding decisions were made by the research team for interpretive analysis of selected cases. Because the framework was used for exploratory interpretation rather than systematic dataset-wide coding, formal intercoder reliability metrics were not calculated.

##### Comparative Contextual Evaluation

Finally, we compared the interpretive patterns of AutoML and PaLM 2 with human rationales. This comparative evaluation highlighted where models aligned with or diverged from human thematic judgments and identified specific narrative features that posed recurrent challenges. The analysis provides insights into the strengths and limitations of each model’s contextual reasoning as reflected in generated outputs in the domain of mental health narratives.

## Results

### Overview

This section presents the results of comparing model outputs with human judgment, detailing their predictive performance, agreement patterns, and interpretability characteristics.

### AI Models’ Internal Performance on MH-RAC

As shown in Table 1, AutoML demonstrated strong performance on the internal test set across all labels, achieving an overall area under the precision-recall curve (AUPRC) of 0.93, with micro-averaged precision, recall, and *F*_1_-scores of 0.86. Label-level results showed the highest performance for the drugs and alcohol category (AUPRC=0.99; *F*_1_-score=0.96), followed by early life (AUPRC=0.94; *F*_1_-score=0.83) and personality (AUPRC=0.90; *F*_1_-score=0.86). Performance was comparatively lower for the trauma and stress category (AUPRC=0.86; *F*_1_-score=0.78).

**Table 1.** AutoML evaluation metrics.

Labels	AUPRC^a^	Precision	Recall	*F*_1_-score
All labels	0.93	0.86	0.86	0.86
Drugs and alcohol	0.99	0.96	0.96	0.96
Early life	0.94	0.87	0.80	0.83
Personality	0.90	0.85	0.88	0.86
Trauma and stress	0.86	0.77	0.80	0.78

a AUPRC: area under the precision-recall curve.

**Figure 1.**
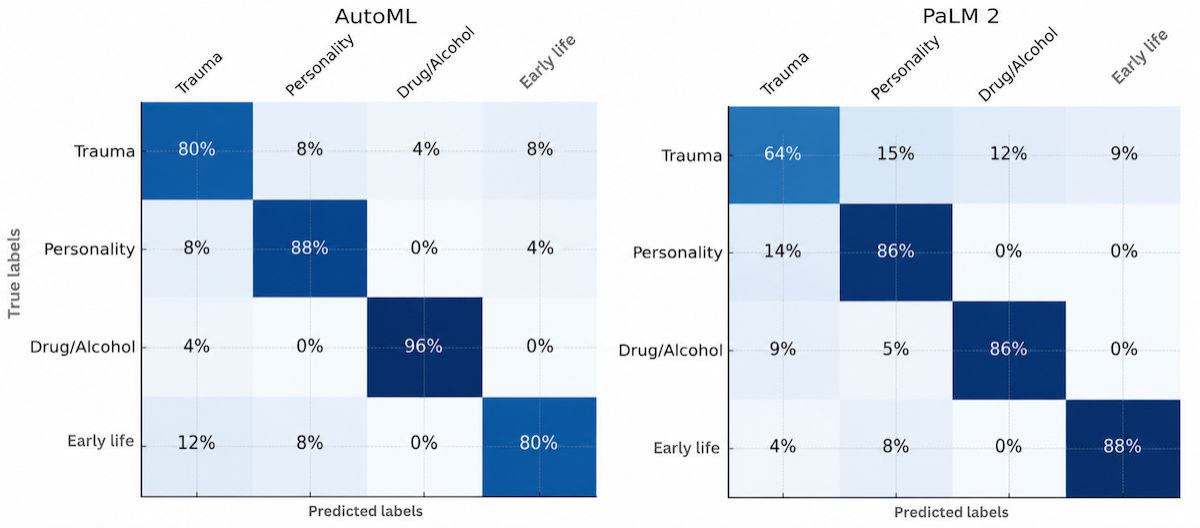
Confusion matrices for AutoML and PaLM 2 showing percentage agreement between true labels (rows) and predicted labels (columns) across 4 mental health root-cause categories.

The confusion matrices (Figure 1) further highlighted error patterns. AutoML classified drugs and alcohol, and personality posts with high accuracy (96% and 88%, respectively), showing minimal confusion. However, it shows moderate confusion in the trauma and stress at 80% accuracy with a misclassification rate of 8% for early life and 8% for personality. Similarly, the early life category sees 80% accuracy, with the model misclassifying 12% of posts as trauma and stress and 8% as personality.

The PaLM 2 model achieved precision, recall, and *F*_1_-score of 79% on its validation set, as shown in Table 2. Label-wise performance ranged from 0.64 *F*_1_-score for trauma and stress to 0.88 *F*_1_-score for early life, indicating that class-specific difficulty patterns were broadly similar to AutoML, with trauma and stress remaining the most challenging category.

Further label-specific performance variability for PaLM 2 is shown in Figure 1. For the trauma and stress category, accuracy was 64%, with misclassifications into personality (15%), drugs and alcohol (12%), and early life (9%). The personality category showed an accuracy of 86%, with 14% misclassified as trauma and stress. For drugs and alcohol, accuracy was 86%, with 9% misclassified as trauma and stress and 5% as personality. The early life category achieved 88% accuracy, with few misclassifications into personality (8%) and trauma and stress (4%).

It is important to note that AutoML and PaLM 2 metrics are derived from different data splits (internal test set vs validation set, respectively) and therefore cannot be directly compared to determine which model demonstrates superior aggregate performance. The definitive comparison of their real-world applicability is their performance against human consensus on the completely unseen set of 50 posts, which is presented in the following sections. Additionally, the 2 models differ fundamentally in their training regimes, so comparisons should be interpreted cautiously.

**Table 2.** PaLM 2 validation metrics.

Labels	Precision (%)	Recall (%)	*F*_1_-score
All labels	79	79	0.79
Drugs and alcohol	86	86	0.86
Early life	88	88	0.88
Personality	86	86	0.86
Trauma and stress	64	64	0.64

### Models Performance on Unseen RMHD-Seed

We evaluated both models on RMHD-Seed using the human consensus as the reference standard. As shown in Multimedia Appendix 1, PaLM 2 correctly matches 40 out of 50 (80% concordance) human consensus labels. In comparison, AutoML correctly labeled 34 out of 50 (68% concordance) posts. This difference reflects variation in model concordance with human consensus on unseen narratives.

To examine whether this accuracy difference was statistically significant, we performed McNemar test on the paired classification outcomes, coding each model’s prediction as correct or incorrect relative to the consensus label. Of the discordant predictions, PaLM 2 correctly classified 12 posts that AutoML misclassified, while AutoML correctly classified 6 posts that PaLM 2 misclassified.

The difference was not statistically significant (*χ*²_₁_=1.39, *P*=.24), indicating that although the models differed in their interpretive patterns, there was no evidence of a difference in paired classification accuracy on this dataset.

### Interrater Reliability Analysis

**Figure 2.**
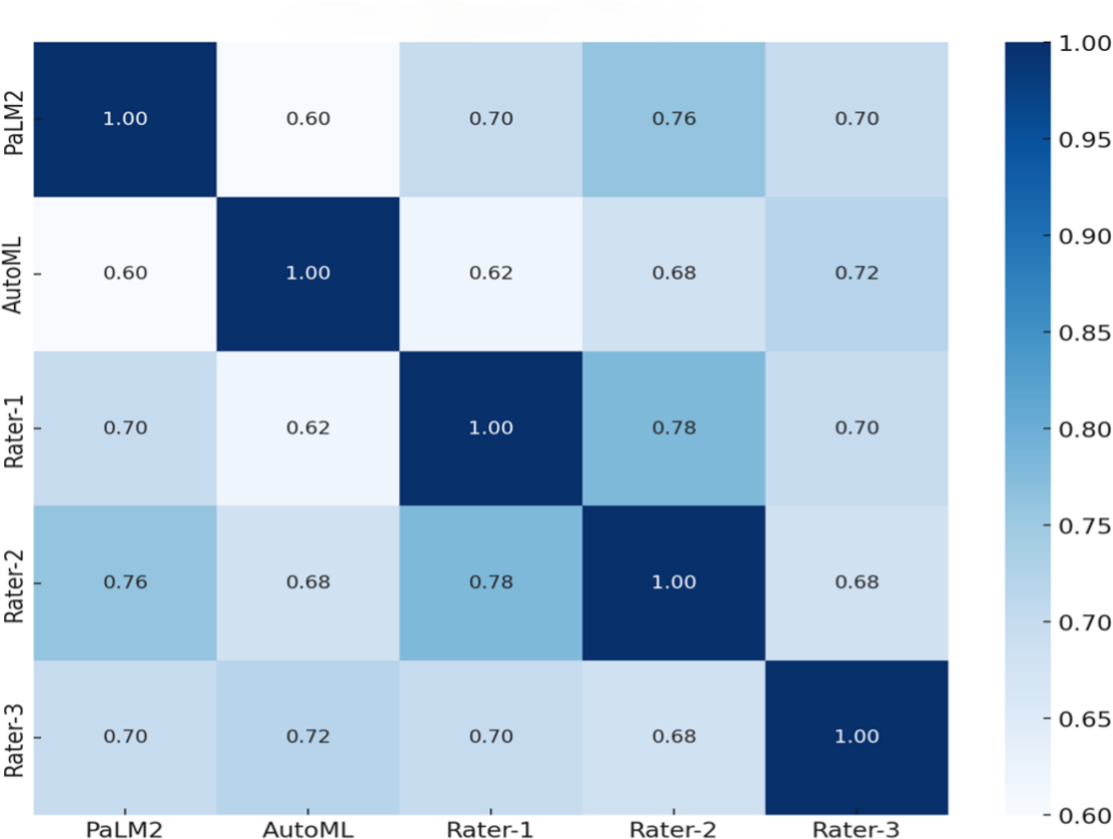
Cohen κ rater agreement matrix among PaLM 2, AutoML, and 3 human annotators.

We next assessed agreement among human raters and between humans AI models using MHEC. Cohen κ (Figure 2) indicated substantial agreement among human annotators (Cohen κ=0.68‐0.78). PaLM 2 demonstrated a similar level of agreement with human raters (Cohen κ=0.70‐0.76), whereas AutoML showed slightly lower levels of agreement (Cohen κ=0.62‐0.72). Agreement between the 2 AI models was the lowest (Cohen κ=0.60), suggesting model-specific decision patterns and distinct sources of error.

### Misclassification Pattern

This section reports misclassification patterns observed across both models, including overall error distributions, label confusions, and label-specific difficulties.

### Overall Misclassification Burden

#### Overview

**Figure 3.**
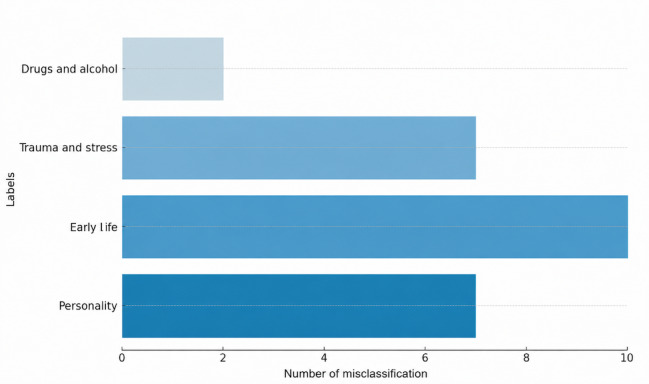
Total number of misclassifications by labels for both models.

The aggregated misclassification burden across both models is shown in Figure 3. Early life accounted for the highest combined number of misclassifications (n=10), followed by trauma and stress, and personality (n=7 each). Drugs and alcohol showed the lowest error count (n=2). These totals reflect the combined errors produced by PaLM 2 and AutoML on RMHD-Seed posts.

#### Class-Wise Error Distribution

PaLM 2 achieved complete accuracy for the drugs and alcohol category, correctly classifying all 10 posts. For early life, 6 posts were correctly classified and 1 was misclassified as drugs and alcohol. In the trauma and stress category, 16 posts were correctly classified, with the remaining 2 misclassified as personality. The personality category showed the highest number of class-level errors, with only 8 posts correctly classified and the remaining 7 misclassified as early life (n=4) and trauma and stress (n=3).

AutoML correctly classified 9 of the 10 drugs and alcohol posts, with 1 misclassified as personality. For early life, 6 posts were correctly identified and 1 was assigned to trauma and stress. In the trauma and stress category, 11 posts were correctly classified, while misclassifications included 3 assigned to early life and 4 to personality. Within the personality category, AutoML correctly classified 8 posts, while the remaining posts were distributed across drugs and alcohol (n=1), early life (n=3), and trauma and stress (n=3).

Table 3 summarizes class-wise classification performance relative to human consensus across the RMHD-Seed dataset. Personality demonstrated the highest class-level error burden for both models, with PaLM 2 and AutoML each correctly classifying 8 of 15 (53%) posts. Trauma and stress showed the greatest difference between models, with PaLM 2 correctly classifying 16 of 18 (89%) posts compared with 11 of 18 (61%) posts for AutoML. In contrast, drugs and alcohol demonstrated the strongest class-wise agreement across both models.

**Figure 4.**
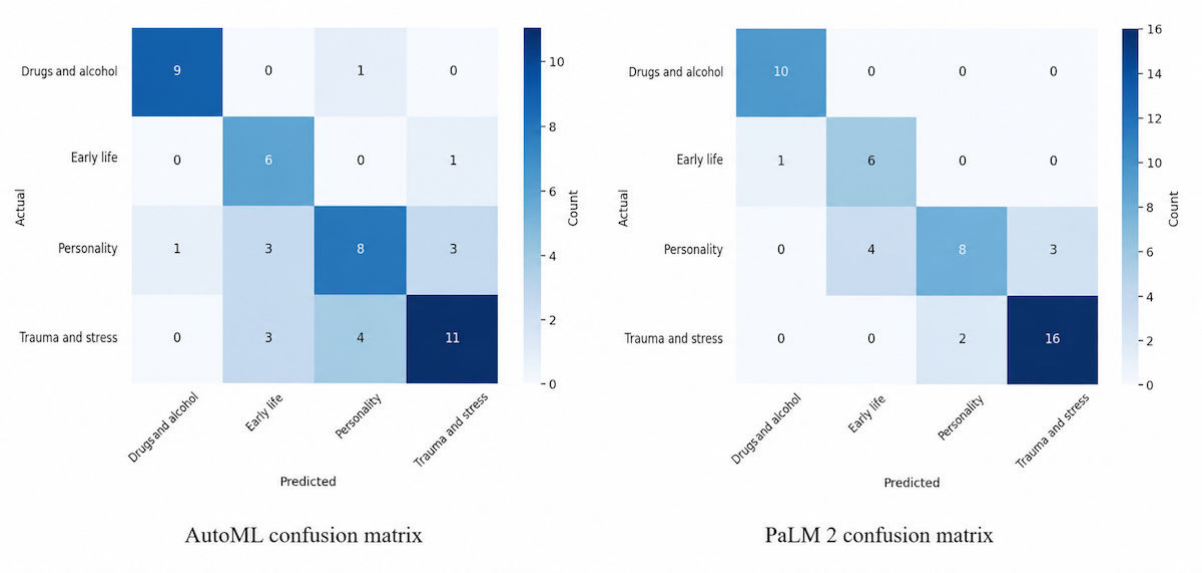
Confusion matrices for AutoML and PaLM 2 showing the distribution of misclassification counts across mental health root-cause labels.

Confusion matrices for each model (Figure 4) further highlight where predictions diverged from human consensus labels.

**Table 3.** Class-wise classification performance relative to human consensus on unseen RMHD-Seed^a^.

Class	Consensus	AutoML correct, n (%)	PaLM 2 correct, n (%)
Personality	15	8 (53)	8 (53)
Early life	7	6 (86)	6 (86)
Trauma and stress	18	11 (61)	16 (89)
Drugs and alcohol	10	9 (90)	10 (100)

a RMHD: Reddit Mental Health Dataset.

PaLM 2 achieved complete accuracy for the drugs and alcohol category, correctly classifying all 10 posts. For early life, 6 posts were correctly classified and 1 was misclassified as drugs and alcohol. In the trauma and stress category, 16 posts were correctly classified, with the remaining 2 misclassified as personality. The personality category showed the highest number of class-level errors, with only 8 posts correctly classified and the remaining 7 misclassified as early life (n=4) and trauma and stress (n=3).

AutoML correctly classified 9 of the 10 drugs and alcohol posts, with 1 misclassified as personality. For early life, 6 posts were correctly identified and 1 was assigned to trauma and stress. In the trauma and stress category, 11 posts were correctly classified, while misclassifications included 3 assigned to early life and 4 to personality. Within the personality category, AutoML correctly classified 8 posts, while the remaining posts were distributed across drugs and alcohol (n=1), early life (n=3), and trauma and stress (n=3).

These patterns indicate that both models most frequently confused trauma and stress, and personality narratives, reflecting the overlap between emotional distress, developmental experiences, and enduring personality-related themes within mental health narratives.

Due to the limited sample size per class (range n=7‐18), per-class statistical significance testing (eg, class-wise McNemar tests) was not conducted. The descriptive breakdown is therefore presented for comparative transparency rather than inferential comparison between models

### Label Difficulty Model-Wise

#### Overview

**Figure 5.**
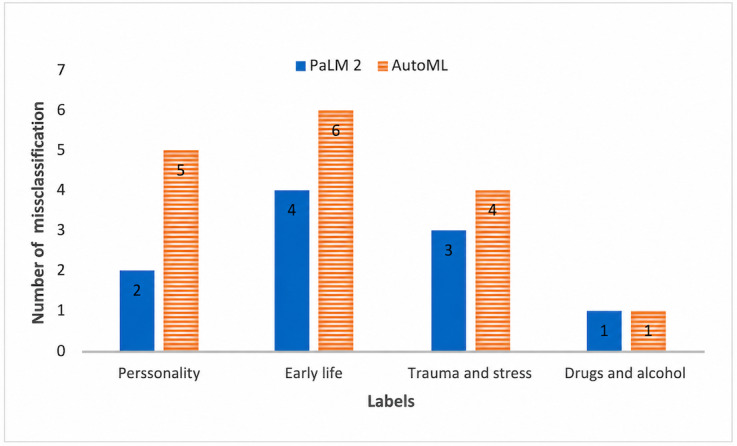
Comparison of misclassification counts by AutoML and PaLM 2 across mental health root-cause categories.

Figure 5 compares the number of misclassifications each model produced for each label. Both models demonstrated substantial difficulty with the early life, with PaLM 2 generating 4 misclassifications and AutoML 6. For personality, PaLM 2 made 2 errors and AutoML made 5. In the trauma and stress category, PaLM 2 misclassified 3 and AutoML misclassified 4 posts. Both models showed minimal difficulty with the drugs and alcohol category, with PaLM 2 and AutoML producing 1 misclassification each.

These results demonstrate the models’ struggle in distinguishing overlapping content in the early life, personality, and trauma and stress categories.

#### Contextual Analysis

To better understand how model predictions related to human interpretations, we conducted a qualitative content analysis of selected RMHD-Seed posts.

#### Qualitative Content Analysis

Textbox 3 presents 3 illustrative instances that were used to examine the narrative features influencing classification outcomes. Table 4 shows the corresponding labels assigned by PaLM 2, AutoML, and the 3 human raters.

Textbox 3. **Reddit post excerpts for rationale reflection (instances 1, 2, and 3) corresponding to Table 4 labeling.**

- **Instance 1:** My poor mental health has been ruining my life for the past three years and I’m so tired of it. Ever since I became pregnant with my second child, I haven’t been myself. I lost a lot of my motivation and desire to do much of anything and apart from rare moments, it hasn’t really changed. I went to therapy and that helped me get out of a desperate place. I’m on meds but still feel numb. Also gained weight and can’t find the motivation to diet and exercise like I did before. This past year I tried going back to working full time in the hopes that it would help me feel like myself, but I couldn’t handle the job and had to quit. Now I’m just sitting on my couch surrounded by a messy house and it’s hard not to hate myself. I don’t have the energy to clean it or play with my kids. I’m afraid my husband is going to start resenting me. After three years it’s hard to believe I’ll ever just snap out of it and be normal again. I don’t have any real reason to feel this way. I’m just mentally weak*.*
- **Instance 2:** I’m a freshman in high school and its already so challenging for me. I don’t know how to talk to my counselor and everyday gets harder. The big exams are a week away and I don’t understand anything. I’m really considering killing myself but the only thing thats holding me back is fear and the sake of my mother because I know she’d be heartbroken. I’m thinking about overdosing but it’s probably not enough. I just don’t want to put a burden on my mom anymore.
- **Instance 3:** That I’m not okay, that everytime I feel remotely motivated I destroy all of it because I feel as if I don’t deserve it. That I don’t deserve to have any chances at living. My exams are next month and shes leaving in a few months for Uni.I’m so lost and I hate myself all the time. I suck at studying and 99% of my assignments aren’t done. I reached out to my friend but I never told him the full extent of my situation. Any time I feel extra bad I get so tempted to cut and hurt myself because I feel like I *deserve* it. My mom is out of the question since I’d hate for her to think my sister influenced me with her own problems (she went through some stuff.I don’t know what I’m doing. My siblings had guidance for their studies when they were my age but I don’t thanks to the stuff happening in the world + I doubt mom wants to pay for extra tutoring for her sad excuse of a kid. I’m lost. I’m scared. I can’t even speak my own language well despite living in the country. Any advice will be helpful.

**Table 4.** Reddit posts labeling by AI models and human raters (instances 1, 2, and 3).

Instance	PaLM 2	AutoML	Rater 1	Rater 2	Rater 3
1	Personality	Trauma and stress	Trauma and stress	Personality	Trauma and stress
2	Trauma and stress	Early life	Trauma and stress	Trauma and stress	Trauma and stress
3	Personality	Early life	Personality	Personality	Personality

In instance 1, the trauma and stress label was linked to an event-driven narrative, where the psychological impact of pregnancy was framed as a traumatic experience with postnatal depression. References to sustained stress and emotional burden supported this interpretation.

In the same narrative, the personality label was justified by traits such as persistent self-criticism and motivational decline. These features could be interpreted as reflecting enduring personality-related traits rather than situational stressors. This example illustrates how a single narrative may support multiple plausible interpretations, complicating the assignment of a single dominant root cause.

These resulted labels in Table 4 highlight the difficulty of disentangling behaviors and expressions associated with trauma-related experiences from those associated with personality-related themes. Both AI models and human raters at times struggled to isolate a single dominant factor driving the narrative.

#### Interpretability Coding and Comparative Model Analysis

As described in the “Methods” section, we applied the 5-code interpretability schema to 3 instances in Textbox 3. These cases were selected based on the labels in Table 4, which illustrated 3 distinct model behaviors: (1) AutoML correct and PaLM 2 incorrect, (2) PaLM 2 correct and AutoML incorrect, and (3) PaLM 2 aligning with human consensus in a personality-based case while AutoML misclassified it as early life, highlighting differences in how model predictions aligned with human consensus labels. Table 5 summarizes the dominant interpretability codes, highlighting differences in how model predictions aligned with human consensus labels.

**Table 5.** Interpretability coding schema.

Instance	Dominant interpretability codes (PaLM 2)	Dominant interpretability codes (AutoML)
1	Context integration: reflected postpartum change, exhaustion, job loss, and emotional distress. Ambiguity and overlap handling: confusion due to competing cues. Causal reasoning: placed greater weighting on self-blame and chronic depletion.	Keyword reliance: anchored to “mentally weak” and “hate myself” pregnancy terms. Weak context integration: underweighted emotional and situational cues. Causal reasoning: used surface patterns rather than narrative synthesis.
2	Context integration: reflected academic pressure, exam stress, and emotional overload. Emotional cue recognition: detected suicidal ideation. Causal reasoning: placed greater emphasis on acute situational stress over developmental context.	Keyword reliance: focused on “freshman” and “school.” Weak emotional cue recognition: underweighted suicidal and crisis cues. Incorrect causal reasoning: associated the narrative with age markers.
3	Context integration: reflected chronic self-blame, identity distress, and self-harm impulses. Emotional cue recognition: captured persistent low self-worth. Causal reasoning: associated the narrative with enduring trait instability. Ambiguity and overlap handling: differentiated trait-level distress from situational stress.	Keyword reliance: overfocused on “Uni,” “siblings,” and “kid.” Weak context integration: showed limited narrative alignment with chronic self-hate and identity fragmentation. Incorrect causal reasoning: associated distress with developmental factors rather than personality-based factors.

In the second instance, which describes academic pressure and suicidal thoughts in a high school student, PaLM 2 classified the post as trauma and stress, consistent with the human consensus. The model outputs appeared to integrate contextual elements such as acute academic distress and references to self-harm, aligning with the thematic cues used by humans.

In contrast, AutoML predicted early life, seemingly influenced by age-related terms such as “freshman in high school” while underweighting crisis indicators. This pattern was consistent with the interpretability coding as PaLM 2 reflected stronger context integration and emotional cue recognition, whereas AutoML relied more heavily on keyword matching. Taken together, these illustrative cases demonstrate variation in feature use across models when narratives contain overlapping or ambiguous root-cause cues. These observations are descriptive in nature and do not imply inferential superiority of one model over another.

## Discussion

### Interpretation Complexities of Mental Health Narratives

This study highlights a distinction between model performance on internal benchmarks and behavior observed when applied to real-world mental health narratives. Although both models performed well during internal evaluation, their performance on 50 unseen posts revealed different concordance patterns relative to human consensus. PaLM 2 achieved 80% concordance with human consensus, compared with 68% for AutoML. These findings suggest that strong benchmark performance does not necessarily correspond to stronger alignment with human interpretation in complex mental health narratives, where contextual and emotionally layered language may influence classification behavior. This section discusses how the models and human raters interpreted mental health narratives, with a focus on misclassification patterns and interpretability.

### Understanding Model Fit and Linguistic Challenges

Both models encountered difficulties with the linguistic complexity of mental health narratives, particularly when posts contained ambiguous, metaphorical, or multilayered descriptions of distress. Narratives often blended formative experiences, ongoing stressors, and enduring personality traits, creating overlap between the early life, personality, and trauma and stress categories. These overlaps challenged not only the models but also human raters. Effective AI models must move beyond literal lexical matching and account for the emotional and situational cues embedded in human narratives.

AutoML’s performance appeared more sensitive to isolated lexical cues, whereas PaLM 2 reflected a broader incorporation of contextual and emotional signals across narratives. However, both models struggled when posts spanned multiple psychological dimensions or expressed distress indirectly. Addressing these challenges will likely require training on larger and more linguistically diverse corpora that better reflect the variety of crisis-related and noncrisis expressions present in mental health discourse.

### Interrater Reliability

The interrater reliability analysis showed substantial agreement among human annotators and indicated that PaLM 2 reached a level of agreement with individual raters comparable to that observed among humans themselves. AutoML demonstrated moderate-to-substantial agreement with greater variability across raters. These findings describe variation in alignment patterns rather than inferential differences between models.

Agreement between PaLM 2 and AutoML was lower (Cohen κ=0.60), indicating that the models emphasized different linguistic features and contextual signals during classification. This divergence was reflected in their misclassification patterns and highlights the importance of evaluating AI systems not only on aggregate performance metrics but also on the nature of their alignment with human annotations across complex narrative data.

### Statistical Analysis

McNemar test of paired classification outcomes did not indicate a statistically significant difference between PaLM 2 and AutoML on unseen posts (*χ*²_₁_=1.39, *P*=.24). This result should be interpreted cautiously considering the limited evaluation sample (n=50), which constrains statistical power and may limit the ability to detect small differences between models.

Descriptive differences were observed in the distribution of discordant cases, with PaLM 2 correctly classifying 12 instances where AutoML misclassified, and AutoML correctly classifying 6 instances where PaLM 2 misclassified. However, these patterns do not support inferential claims regarding model superiority. Instead, they highlight the importance of combining quantitative performance metrics with qualitative analyses to better understand model behavior, particularly when sample sizes are modest.

### Interpreting Misclassification Patterns

Despite relatively high accuracy, both AI models and human raters exhibited misclassification trends, particularly in the early life category (Figure 5). This label was often difficult to distinguish from personality and trauma and stress because narratives describing formative experiences frequently intertwined with enduring dispositions or current stressors. By contrast, the drugs and alcohol category was more straightforward, as it tended to contain explicit references to substance use.

AutoML frequently misclassified early life narratives as trauma and stress or personality, consistent with a heavier reliance on literal or structural language markers and a weaker integration of context. PaLM 2 often reflected stronger contextual alignment across narrative elements but sometimes conflated long-term developmental experiences with personality-related emotional patterns.

The first instance in Textbox 3 illustrates this overlap. The post describing postnatal depression and loss of motivation includes both situational stressors and self-perceived traits such as “mentally weak” and “hate myself.” Two human raters and AutoML classified it as trauma and stress, whereas PaLM 2 and another human rater labeled it as personality. This divergence underscores how subtle linguistic cues like “motivation,” “weakness,” and “hate myself” can shift interpretation from situational distress to intrinsic disposition.

The confusion matrices (Figure 4) further demonstrate that human raters and AI models face similar ambiguity when differentiating developmental context from enduring personality features. This shared confusion highlights the inherent complexity of mental health narratives and reinforces the need for models to integrate deeper semantic and contextual representations rather than relying on surface-level lexical cues.

### Interpreting Human-AI Consensus

The MHEC analysis also reveals 21 instances where human annotators and AI models agree on the root cause labels. This consensus is typically achieved when narratives contain clear and impactful keywords. For example, the first instance in Textbox 4 describes clear distressing expressions of early life events such as parental divorce, financial instability, and explicit suicidal thoughts. The specificity and clarity of these cues enabled both models and human raters to categorize the narrative as early life, as shown in Table 6.

Textbox 4. **Reddit post excerpts for rationale reflection (instances 1 and 2) corresponding to Table 6 labeling.**

- **Instance 1:** “my life was nice before my parents divorced. while growing up I was very insecure about myself, my family is poor because my father doesn’t pay his debts which are in total most than half a million € and now my mother has to take care of them. I used to be a great student but I just dont f care now, I feel like im irrelevant and I constantly think about suicide. If I dont do it now i will do it soon, i have thought about shooting at my head with a gun which i think would be the less painful. [Some text omitted for brevity]”
- **Instance 2:** “Every day to me is being a constant torture. I’m sleeping 14 hours a day, hoping some-day i never wake up again. I’ve lost 130 k USD on online casino (started with 12 k USD, all my cryptocurrency reserves) in February, i’m always thinking myself i could’ve just stopped and call done, but that wasn’t possible since i only got this far by risking it all. Gambling addiction is hell. I never had a job, i have no skills, i’m from a shithole country, national minimum monthly wage here is equivalent to 300 USD. Thankfully to cryptocurrency and NFT games, i made a good money being at right time and place. I was very lucky. But its all gone.I have 2 choices: either suffer everyday, relapsing to gambling addiction and blaming myself for the rest of my life or cease for all.[Some text omitted for brevity]*”*

**Table 6.** Reddit posts labeling by AI models and human raters (instances 1 and 2) reflect human AI consensus.

Instance	PaLM 2	AutoML	Rater 1	Rater 2	Rater 3
1	Early life	Early life	Early life	Early life	Early life
2	Trauma and stress	Trauma and stress	Trauma and stress	Trauma and stress	Trauma and stress

The second instance in Textbox 4 also shows consensus on gambling addiction, where detailed descriptions of financial ruin and emotional despair match predefined guidelines for traumatic life events. These examples emphasize the importance of clarity and specificity in language, suggesting that enhancing AI sensitivity to these features improves overall interpretive accuracy.

### Interpreting Model Reasoning Patterns

The interpretability analysis revealed distinct decision patterns for AutoML and PaLM 2 as summarized in Table 5. AutoML’s predictions were mostly driven by Keyword Reliance. This led to misclassifications when emotionally charged terms (eg, “school,” “freshman,” and “hate myself”) were present without sufficient contextual integration. These instances illustrate limitations in handling narratives where surface cues do not reflect the dominant psychological theme.

In contrast, PaLM 2 reflected the incorporation of a broader range of contextual features across multisentence narratives. However, PaLM 2 was not exempt from error. Its misclassifications often reflected difficulty in “Ambiguity and Overlap Handling.” For example, in the first instance of Textbox 3, the model outputs incorporated multiple cues such as exhaustion, self-blame, and postpartum decline. However, it appeared to place greater emphasis on the dominant causal factor, favoring a “Personality” interpretation over situational postpartum stress. Such patterns resemble challenges observed in human labeling when narratives plausibly support more than one root-cause category.

Overall, the interpretability findings suggest that AutoML outputs more frequently reflected surface-level lexical anchoring, whereas PaLM 2 outputs more often demonstrated broader contextual synthesis. These observations provide a descriptive account of how the models’ outputs differed across complex cases. They should not be interpreted as direct evidence of internal model mechanisms or as inferential claims of superiority.

### AI-Human Alignment and Divergence

The comparison between model outputs and human annotations revealed differences in how predictions aligned with consensus labels across specific narrative contexts. These differences were most apparent in posts containing overlapping psychological dimensions or indirect expressions of distress.

A representative example illustrated this pattern in the second instance of Textbox 3. PaLM 2 classified the post as trauma and stress consistent with human consensus. The prediction reflected the presence of contextual indicators such as academic pressures and references to suicidal ideation, which were also emphasized by human raters when determining the dominant root cause.

In the same instance, AutoML assigned an Early Life label, corresponding to the presence of isolated developmental references (eg, “freshman in high school”). This divergence illustrates differences in the types of textual features emphasized by each model, with AutoML placing greater weight on salient lexical cues and PaLM 2 reflecting the incorporation of a broader range of contextual signals.

Across examined cases, divergence from human consensus was more likely when narratives expressed multiple psychological themes or when the dominant causal factor was conveyed implicitly rather than through explicit keywords. These observations highlight variation in feature use across models rather than differences in inferential capability.

### The Explainability-Performance Trade-Off: Interpretability Patterns in Narrative Classification

The comparative evaluation of AutoML and PaLM 2 highlights differences in how models handle contextual information when applied to complex mental health narratives. Although both models performed well on structured internal datasets, their interpretive behavior differs when processing psychologically nuanced, real-world text.

### AutoML: Lexical Feature Reliance

AutoML primarily relies on salient lexical markers over narrative context. This approach leads to misclassifications when surface cues conflict with deeper emotional or situational signals. This pattern is observable in posts where developmental references appear alongside acute distress indicators. These errors reflect an associative reasoning process that lacks the contextual flexibility needed for transparent interpretation in mental health settings.

### PaLM 2: Contextual Feature Integration

In contrast, PaLM 2 exhibited a greater tendency to incorporate multiple narrative elements when forming predictions. Even when incorrect, PaLM 2’s interpretations tended to be thematically coherent and aligned with human reasoning challenges, such as difficulty in prioritizing overlapping root causes. For example, in Instance 1 of Textbox 3, the model outputs reflected the incorporation of multiple relevant cues, such as self-blame, exhaustion, and postpartum decline, and generated a personality label, mirroring one human rater’s view. Although the consensus classified the narrative as trauma and stress, PaLM 2’s misclassification remained understandable within a human interpretive framework.

These observations highlight an interpretability trade-off rather than a performance hierarchy. Differences between models arise not only from prediction outcomes but from the types of features emphasized during classification.

For tasks involving emotionally complex self-narratives, models that incorporate broader contextual signals may offer interpretive insights complementary to models that prioritize lexical associations. Importantly, these findings are descriptive and do not imply inferential superiority of one model over another.

### Implications and Directions for Future Research

Our findings highlight the need for AI models capable of representing the linguistic and contextual complexity of human narratives. This study contributes to digital mental health informatics by demonstrating how AI can assist in identifying potential root-cause patterns in user-generated content. While not intended for clinical diagnosis, this work may inform future applications in digital triage and population-level monitoring.

The qualitative analysis highlights ongoing challenges for AI in processing complex human narratives, particularly in distinguishing situational stressors from enduring Personality traits and disentangling overlapping psychological constructs. Continued improvements in data quality, annotation depth, and model design will be important for enhancing contextual robustness in narrative classification systems.

Future research should prioritize the development of larger and more diverse annotated datasets capturing a wide range of linguistic, emotional, and cultural expressions. Such datasets may improve model sensitivity to subtle narrative cues and reduce the risk of misinterpretations. Another important direction is the development of models capable of integrating temporal and contextual signals such as life-history indicators, evolving stress trajectories, and emotional undertones to support richer and more contextually relevant interpretations.

This study also reinforces the importance of explainable AI for digital mental health contexts. By applying a qualitative interpretability coding framework, we demonstrate how models differ in feature emphasis and classification patterns. Transparent evaluation approaches that consider both performance metrics and interpretability characteristics can support responsible assessment of AI systems in public health and digital care environments.

### Limitations

This study has several limitations. First, RMHD is derived from publicly available, anonymized Reddit posts, which restricts access to demographic and geographic metadata. Reddit’s user base is self-selected, younger, and predominantly English-speaking. These factors introduce demographic and cultural biases that may limit generalizability. Mental health expressions vary across cultures in language, metaphor use, emotional tone, and help-seeking norms. As a result, both models may underperform when applied to other populations. Although we tried to mitigate some risks by focusing our annotation on broad psychological themes rather than culturally specific idioms, this does not eliminate deeper cultural-linguistic bias. For example, some communities emphasize spiritual framing, somatic complaints, or indirect emotional language that may be underrepresented in RMHD. Both PaLM 2 and AutoML may therefore misinterpret culturally patterned expressions of distress when applied beyond English-language online communities.

Second, our annotated dataset (n=800) is also relatively small and focused on 4 broad root-cause categories. The trauma and stress category encompasses a wide variety of issues, including personal, financial, and work-related stressors. Expanding the taxonomy to include more context-specific categories and building larger, multilingual corpora would help capture a broader range of psychological experiences.

Third, although we used the McNemar test to compare model performance on the RMHD-Seed set, the small sample size (n=50) limited statistical power and may have reduced the ability to detect meaningful differences between models. The primary contribution of this study, therefore, lies in its combined quantitative and qualitative perspective rather than the definitive statistical superiority of one model over another. Future studies with larger test sets and CI estimation are needed to support stronger inferential claims.

Fourth, internal performance metrics for AutoML and PaLM 2 were obtained from different internal data splits (AutoML’s internal test set vs PaLM 2’s validation set). Beyond data splits, the models also differ in training regimes, including architecture and pretraining, further limiting direct comparability. This limits direct comparability of their precision and recall values. The primary comparison therefore relies on their performance against human consensus on the 50 unseen RMHD-Seed posts.

Finally, although a human consensus benchmark was used to enhance reliability, it still reflects subjective judgments of a small group of raters. Moreover, the interpretability framework was developed for this study and applied descriptively to selected cases without formal reliability testing. Future research should incorporate larger evaluation sets, broader rater panels, and expanded validation of interpretability methods.

### Responsible AI Statement

This research adheres to JMIR AI’s principles of fairness, transparency, and accountability. Transparency is ensured by reporting all model configurations, evaluation procedures, and interpretability methods. Fairness considerations were addressed by identifying potential demographic and linguistic biases in both the dataset and the human evaluation process, including the limited rater diversity. Accountability was maintained by restricting the study to exploratory analysis, applying human oversight throughout, and clearly outlining the limitations and validation requirements needed before any future applied use.

### Conclusions

This study examined how AutoML and PaLM 2 processed and classified the root causes in mental health narratives on social media. Evaluating model predictions against human consensus and analyzing interpretability patterns showed that internal benchmark performance alone is insufficient for understanding model behavior on complex, real-world text. Differences were observed in how each model’s predictions aligned with human annotations on unseen posts and in the types of contextual features emphasized during classification.

The multilayered evaluation, including interrater reliability, error profiling, and qualitative reasoning analysis, revealed that differences between the models extend beyond accuracy and reflect distinct decision strategies. These findings highlight the value of combining quantitative and qualitative assessments when evaluating AI systems for mental health–related text analysis.

Although limited by dataset size, rater diversity, and the subjectivity of consensus labels, this study contributes to mental health informatics by demonstrating the importance of interpretability-focused evaluation. Approaches that explicitly examine contextual feature use and decision transparency can support more responsible assessment of AI systems applied to psychologically complex self-narratives.

## Supplementary material

10.2196/71219 Multimedia Appendix 1 Percentage agreement and disagreement of PaLM 2 and AutoML predictions with human consensus labels.

## Abbreviations

AUPRC: area under the precision-recall curve
MH-RAC: Mental Health-Reddit Annotated Corpus
MHEC: Mental Health Evaluation Corpus
NHMRC: National Health and Medical Research Council
RMHD: Reddit Mental Health Dataset
