# Supplementary figures and images for: AI as the Interpreter for Identifying Root Causes and Emotional Themes in Mental Health Narratives on Reddit Using AutoML and PaLM 2: Mixed Methods Study
Source: JMIR AI. 2026 Jul 13;5:e71219. doi: 10.2196/71219 (PMC13361628; doi:10.2196/71219)

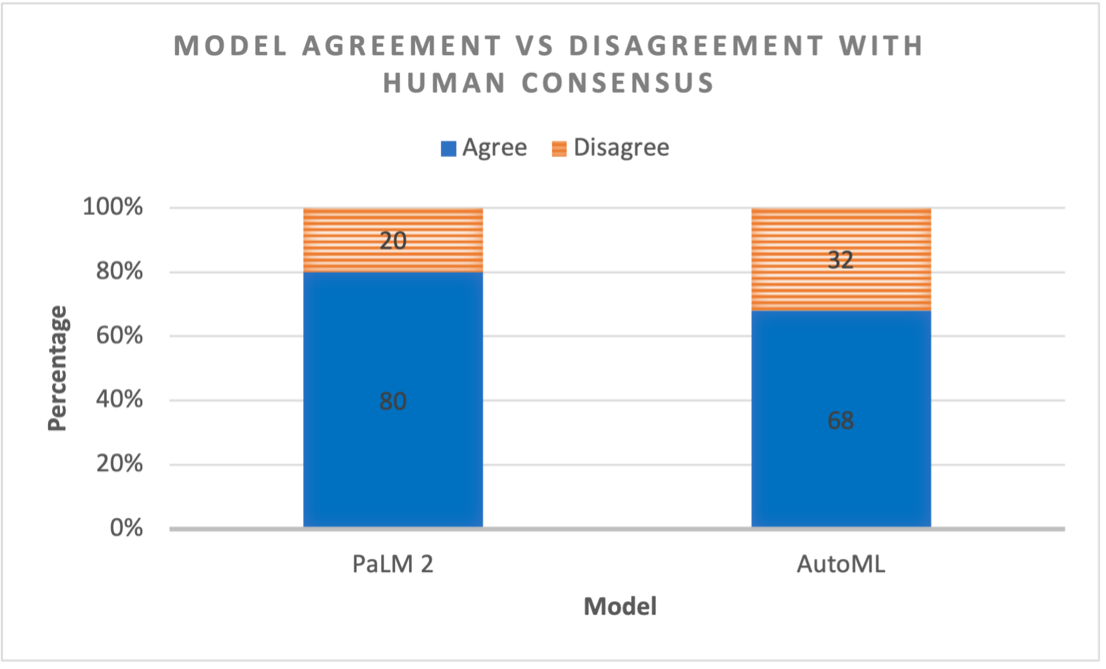

Supplement: Multimedia Appendix 1 [file ai-v5-e71219-s001.png]
